# Reconfigurable Droplet–Droplet Communication Mediated by Photochemical Marangoni Flows

**DOI:** 10.1021/jacs.3c12882

**Published:** 2024-02-23

**Authors:** Anne-Déborah C. Nguindjel, Stan C. M. Franssen, Peter A. Korevaar

**Affiliations:** Institute for Molecules and Materials, Radboud University, Heyendaalseweg 135, Nijmegen 6525 AJ, The Netherlands

## Abstract

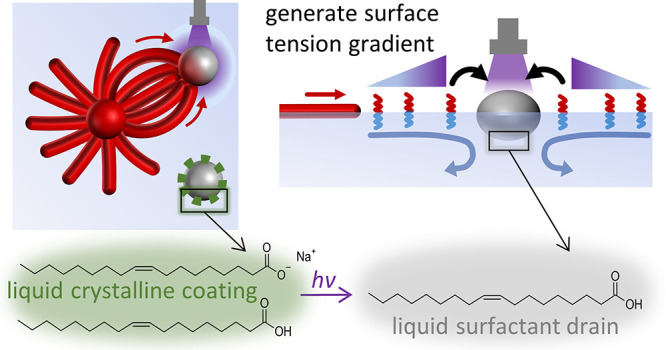

Droplets are attractive building blocks for dynamic matter that organizes into adaptive structures. Communication among collectively operating droplets opens untapped potential in settings that vary from sensing, optics, protocells, computing, or adaptive matter. Inspired by the transmission of signals among decentralized units in slime mold *Physarum polycephalum*, we introduce a combination of surfactants, self-assembly, and photochemistry to establish chemical signal transfer among droplets. To connect droplets that float at an air–water interface, surfactant triethylene glycol monododecylether (C_12_E_3_) is used for its ability to self-assemble into wires called myelins. We show how the trajectory of these myelins can be directed toward selected photoactive droplets upon UV exposure. To this end, we developed a strategy for photocontrolled Marangoni flow, which comprises (1) the liquid crystalline coating formed at the surface of an oleic acid/sodium oleate (OA/NaO) droplet when in contact with water, (2) a photoacid generator that protonates sodium oleate upon UV exposure and therefore disintegrates the coating, and (3) the surface tension gradient that is generated upon depletion of the surfactant from the air–water interface by the uncoated droplet. Therefore, localized UV exposure of selected OA/NaO droplets results in attraction of the myelins such that they establish reconfigurable connections that self-organize among the C_12_E_3_ and OA/NaO droplets. As an example of communication, we demonstrate how the myelins transfer fluorescent dyes, which are selectively delivered in the droplet interior upon photochemical regulation of the liquid crystalline coating.

## 1 Introduction

Reconfigurable connections allow decentralized units within living organisms to communicate and collectively coordinate their organization. For example, slime mold *Physarum polycephalum* grows complex networks of filaments that initially explore their entire surroundings for food, and upon localizing the spots rich in food, it only maintains the shortest pathways to distribute the nutrients efficiently.^1^ Establishing such “embodied intelligence”^2−4^ in synthetic matter requires elementary units that collectively operate as they communicate through adaptive connections.

Droplets provide an attractive design strategy for the development of such self-organizing matter. Their motion, both along air–water interfaces and in aqueous solutions, can be directed via the Marangoni effect: a mass transfer from low toward high surface tension regions.^5−7^ Surface tension gradients enable motile droplets to display chemotaxis,^8−12^ solve mazes,^13,14^ organize into patterns,^15,16^ form dynamic swarms,^17−19^ or display predator–prey behavior.^20^ Furthermore, droplets have acquired an expanding range of applications, varying from sensing,^21,22^ optics,^23^ reconfigurable objects,^24,25^ controlled reactors,^26^ computation,^27−29^ and protocells.^30−35^

**Figure 1:**
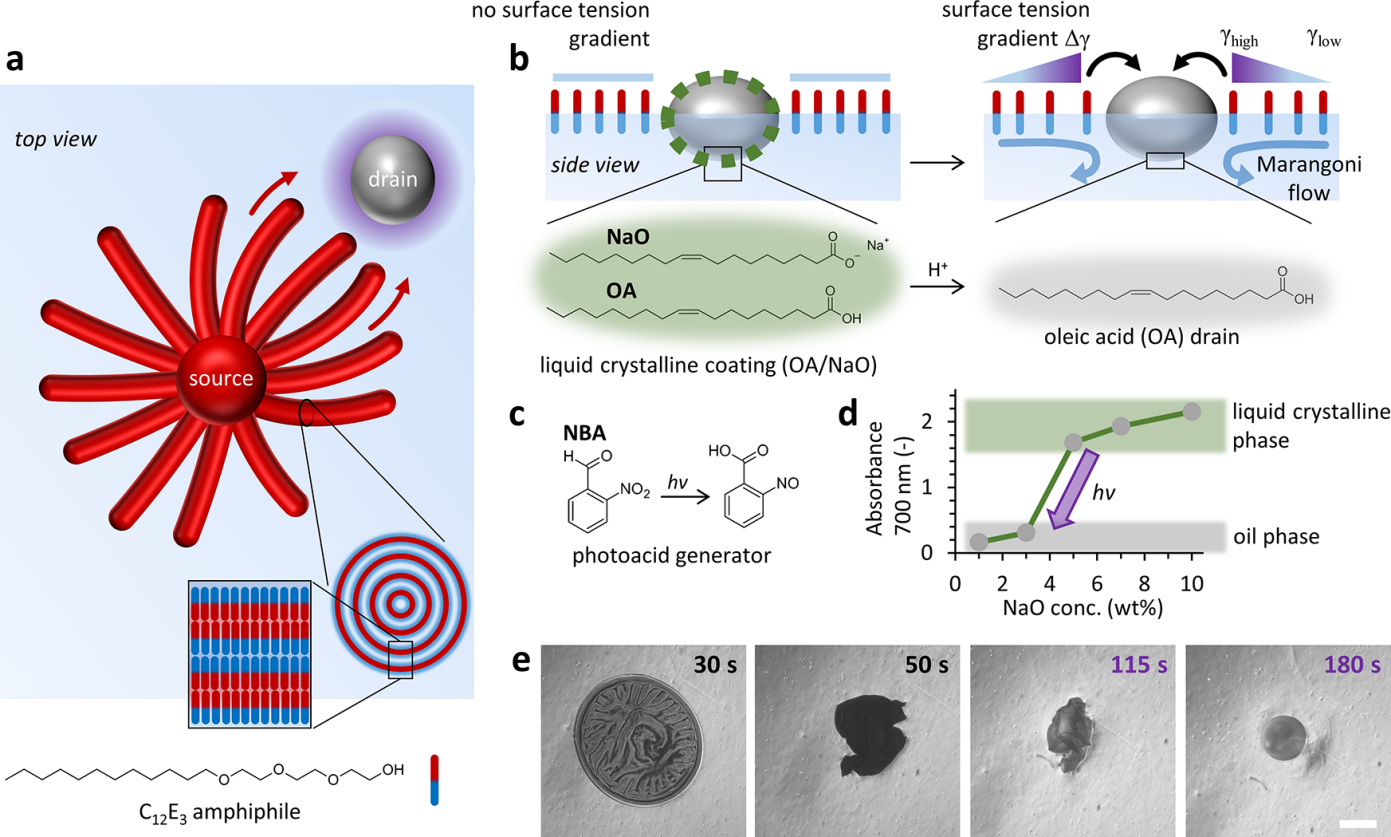
Design of interconnected droplet–filament networks with photocontrolled drain droplets. (a) A source droplet (red sphere) of the C_12_E_3_ amphiphile (red-blue rods) grows multilamellar filaments while floating at the air–water interface. The photoactive drain droplet (gray sphere) attracts the filaments upon generating a surface tension gradient (purple halo). (b) To direct the myelins toward a selected droplet, we develop photoactive drain droplets (gray sphere) based on mesitylene (mesi), oleic acid (OA), and sodium oleate (NaO) mixtures. NaO/OA forms, when deposited on an aqueous solution, a liquid crystalline coating at the oil–water interface of the drain droplet (green dashed line). The coating suppresses the uptake of surfactants (i.e., C_12_E_3_) from the air–water interface. Via the photoacid generator 2-nitrobenzaldehyde (NBA, c), exposure of UV generates an acid that protonates NaO and therefore disintegrates the liquid crystalline coating, which enhances the activity of droplet to deplete surfactants, generate a surface tension gradient, and attract a Marangoni flow. (d) The liquid crystalline phase for 1:1 OA/mesi mixtures with water (5:3 volume ratio) is formed with NaO contents ≥5 wt % in the drain solution, as shown by the sharp increase in absorbance at 700 nm. (e) Optical microscopy images of a photoactive drain droplet upon exposure to UV: A 20 wt % NBA drain droplet (1 μL) is deposited at an aqueous solution (0.63 wt % sodium alginate and 100 μM C_12_E_3_) at *t* = 0 s. The drain droplet is exposed to UV from *t* = 109 s; the scale bar represents 1 mm.

Departing from functions that rely on individual droplets, communication among collectively operating droplets allows them to form structures or perform behavior that cannot be established with individual droplets. Chemical communication typically relies on exchange of signal molecules, which however spread upon diffusion throughout the medium and therefore cannot target specific droplets. This prompted us to explore the concept of self-assembled filaments acting as wires that enable transfer of chemical signals from source droplets to drain droplets. As source droplets, we use microliter droplets of the amphiphile compound triethylene glycol monododecyl ether (C_12_E_3_) that grow so-called myelins—the “wires”—when deposited at an air–water interface (Figure 1a).^36,37^ The assembly of myelins starts with the formation of a lamellar phase of closely packed amphiphile bilayers at the boundary of the amphiphile droplet, in contact with water.^38−44^ The spaces in between these bilayers take up more water; the resulting osmotic pressure forces the bilayers to buckle and form myelins of 20–50 μm in diameter and up to multiple millimeters in length that progress over the air–water interface. To direct the growth of these myelins, we have shown earlier how surface tension gradients can be generated upon localized surfactant depletion from the air–water interface by a (hydrophobic) drain droplet, resulting in a Marangoni flow that draws the myelins toward the drain droplet.^45^

In this work, we implement photochemical control over the activity of drain droplets to generate a Marangoni flow and therefore attract these myelin connections (Figure 1b). Light can be used for contactless manipulation of surface tension gradients, typically by utilizing photoswitchable surfactants or phototriggered reactions that lead to surfactant (de)activation.^46−52^ Here, we design a system that encompasses the photoactive compounds in droplets, allowing them to function as individually targetable nodes in the UV-controlled network organization. Next, we explore the reconfigurability of the myelin connections among the droplets, as well as their capability to selectively deliver fluorescent dyes at UV-targeted droplets, providing a first step toward communication.

## 2 Results and Discussion

### 2.1 Design of the Photoactive Drain Droplet System

The design of the photocontrolled drain droplets exploits the liquid crystalline phase that is reported by Mele et al. in the ternary phase diagram for bulk mixtures of water, oleic acid (OA), and sodium oleate (NaO).^53^ When a drain droplet containing OA and NaO is deposited at an air/water (a/w) interface, the liquid crystalline phase at the droplet interface is anticipated to initially suppress the depletion of C_12_E_3_ surfactants from the air–water interface (Figure 1b). Next, a photoacid generator protonates NaO upon UV exposure, disintegrates the liquid crystalline coating at the interface of a drain droplet, and allows for depletion of C_12_E_3_ from the air–water interface—which in turn will drive the attraction of the myelins. We selected 2-nitrobenzaldehyde (NBA)^54−56^ as a photoacid generator that undergoes upon UV exposure an intramolecular rearrangement^57^ into 2-nitrosobenzoic acid (p*K*_a_ = 3.63, Figure 1c).^58^ The limited solubility of NBA in pure OA prompted us to prepare drain droplets based on a 1:1 oleic acid/mesitylene mixture. Mesitylene (mesi) readily dissolves NBA, whereas its low water-miscibility and low density allow the drain droplet to float on the aqueous medium. To suppress the initial C_12_E_3_ uptake at the drain by formation of the liquid crystalline coating, we use a 10 wt % sodium oleate (NaO) concentration in 1:1 v/v mesi/OA; the NBA concentration is varied between 0 and 20 wt %.

We determined the NaO content of the drain that marks the transition from liquid crystalline to liquid phase. OA/mesi/NaO solutions only form a liquid crystalline phase in contact with water when the NaO content is 5 wt % or higher, as evidenced by the sharp increase in UV/vis scattering (Figure 1d). NBA does not affect the capability of oleate to form a liquid crystalline phase (Figure S1). Next, when a 20 wt % NBA drain droplet (1 μL) is deposited at the a/w interface of an aqueous solution of C_12_E_3_ (100 μM) and sodium alginate, it forms a flattened structure that wrinkles and contracts, indicative of a liquid crystalline coating at the oil–water interface (Figure 1e and Movie 1). Upon exposure to UV (370 nm), the drain converts into a spherical droplet within approximately 100 s. Furthermore, probing the absorption bands typical of the nitro group in infrared (IR) spectroscopy reveals the NBA conversion upon UV exposure (Figure S2). Together, these observations imply that photoactivation of NBA protonates the NaO surfactant, which disintegrates the liquid crystalline coating of the drain.

### 2.2 Controlling the Myelin Trajectories with Photoactive Drains

**Figure 2:**
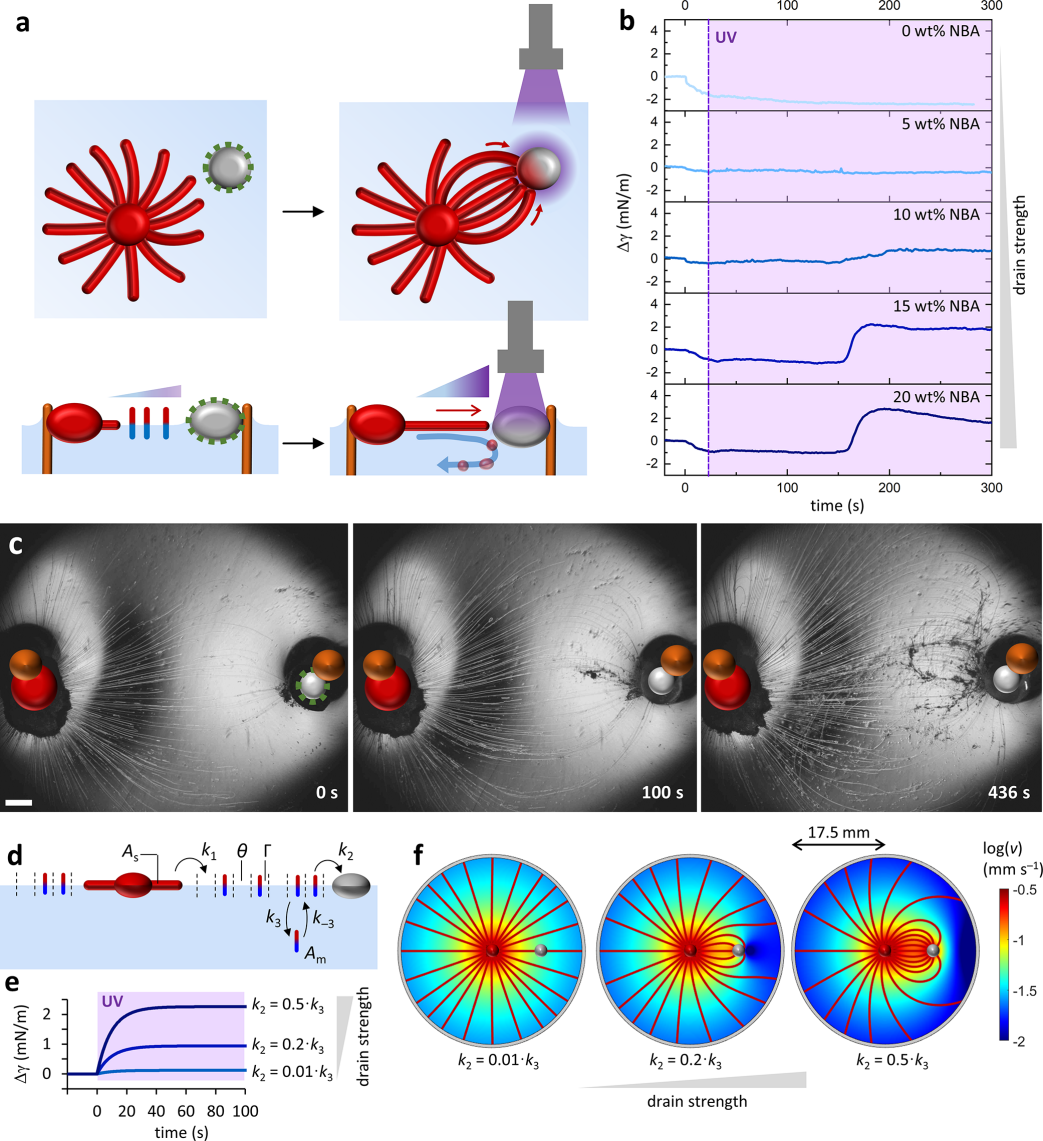
Attraction of the myelin filaments toward a photoactive drain. (a) Scheme representing the attraction of myelins toward the drain due to the phototriggered Marangoni flow. After prolonged UV exposure, part of the myelins get absorbed in the uncoated drain, while others get caught in the Marangoni backflow that pushes them into the bulk of the aqueous medium, away from the drain. (b) Time-dependent change in surface tension Δγ of air–water interfaces of 100 μM C_12_E_3_/0.63 wt % sodium alginate solutions, with a drain droplet (1 μL, containing 0–20 wt % NBA) upon UV exposure. (c) Optical microscopy images showing myelins being attracted toward a 20 wt % NBA drain droplet due to the UV-triggered Marangoni flow (UV is turned on at *t* = 0 s). The scale bar represents 1 mm. (d) Scheme of the kinetic model describing the depletion of surfactants from the air–water interface toward the aqueous phase and the drain. (e) Simulations of the surface tension Δγ for a C_12_E_3_ solution (100 μM) upon UV activation of drain droplets with different *k*_2_ values at *t* = 0 s. (f) Simulated flow velocity profiles among the source (red sphere in center) and drain (gray sphere) for increasing drain strengths from left to right. The red lines indicate the streamlines from the source to drain.

We aim to use the photocontrolled degradation of the liquid crystalline coating of the drain to generate a surface tension gradient that leads to the attraction of the myelins (Figure 2a). First, the capability of the drain droplets to absorb C_12_E_3_ from the a/w interface upon photoactivation was assessed by measuring the change in surface tension of the a/w interface Δγ. Drain droplets with different NBA concentrations are applied at the interface of an aqueous C_12_E_3_ solution (100 μM) with sodium alginate (Figure 2b). Initially, Δγ slightly decreases, which we ascribe to the release of NaO from the droplet being more efficient in equilibrating the surface tension of the a/w interface than the slow adsorption of C_12_E_3_ from the viscous bulk medium. When UV is switched on, Δγ depends on the NBA concentration in the drain: For 0 and 5 wt % NBA, Δγ does not change significantly. This indicates that the depletion rate of surfactant – C_12_E_3_ or NaO – from the a/w interface by the drain is insufficient to outcompete the adsorption of C_12_E_3_ from the bulk medium to the a/w interface. For 10, 15, and 20 wt % NBA, Δγ steadily rises upon UV exposure after a lag time of approximately 120 s, with the increase in Δγ being proportional to the NBA concentration. We rationalize this lag time by the initial presence of the liquid crystalline coating around the drain droplet, which limits the C_12_E_3_ absorption. Only when the NaO concentration drops below 5 wt % upon NBA photoconversion, the coating disappears. For drains with an NBA concentration that is sufficient to reduce the NaO concentration from 10 wt % to <5 wt % (i.e., NBA ≥ 10 wt %), a comparable UV exposure time is required to start the disintegration of their coating. This is most likely because the production of acid at the drain interface is limited by its optical density rather than the NBA concentration. The overall surfactant uptake is determined by the total reduction of NaO in the drain, and therefore, the rise in Δγ is larger with higher NBA concentrations. We note that the extent at which Δγ subsequently declines again is inversely proportional to the initial NBA concentration, implying that a rapid uptake of C_12_E_3_ also results in a faster saturation of the drain droplet.

The stronger depletion of C_12_E_3_ by drain droplets that are loaded with higher NBA photoacid generator contents also results in stronger attraction of myelins. To assess the attraction, we focus on controlling the myelin trajectory in a system where source and drain droplets are both maintained at fixed positions at the meniscus of steel pillars placed in the solution.^59^ The aqueous solution contains sodium alginate to enhance the stability of the myelins.^36^ Upon photoactivation of the drains for several minutes, more myelins curve toward the drain as they progress over the a/w interface, implying an intensification of the Marangoni flow as the absorption of the surfactant at the drain becomes stronger (Figure 2c). After several minutes of UV exposure, a backflow^45^ emerges that carries some of the myelins to the bulk of the solution and away from the drain. The attraction of myelins toward the drain upon photoactivation is most notable for 15 wt % NBA and 20 wt % NBA drains (Figure S3 and Movie 2), in analogy to the rise in surface tension that was observed for these NBA concentrations (Figure 2b).

To further quantify the surfactant depletion at the photoactivated drains, we use a kinetic model that we developed earlier.^45^ The model describes the surfactant release from the source to the a/w interface with rate constant *k*_1_, the depletion from the a/w interface to the drain with rate constant *k*_2_, and the reversible depletion from the a/w interface to the underlying aqueous medium with rate constants *k*_3_ and *k*_–3_ (Figure 2d). Via the Frumkin isotherm, the surface tension can be calculated from the surfactant density Γ.^60^ Next, the surfactant depletion rate from the a/w interface to the aqueous phase (Φ_water_) and the drain (Φ_drain_) allows predicting the velocity profiles of the fluid along the a/w interface toward the regions where the depletion occurs: the boundary of the Petri dish and the perimeter of the drain (see details in the Supporting Information).^61^ Upon activation of the drain (i.e., changing the rate constant *k*_2_ = 0 to *k*_2_ > 0), changes in surface tension are predicted (Figure 2e) that match with the experimental kinetics for 10, 15, and 20 wt % NBA drains shown in Figure 2b. Furthermore, these simulations show that an increase in Φ_drain_ by a factor of 50 is sufficient to generate the raise in surface tension Δγ = 2 mN m^–1^ as observed with 20 wt % NBA. Here, Φ_drain_ increases up to 0.5·Φ_water_ = 5.2 × 10^–13^ mol cm^–1^ s^–1^, which implies a C_12_E_3_ uptake by the drain in the nanoliter range. Increasing *k*_2_ generates more intense surface tension gradients (Figure S4) that attract larger amounts of myelins, visualized as flow lines that curve from the source to the photoactivated drain in Figure 2f.

### 2.3 Competition among Multiple Drains

**Figure 3:**
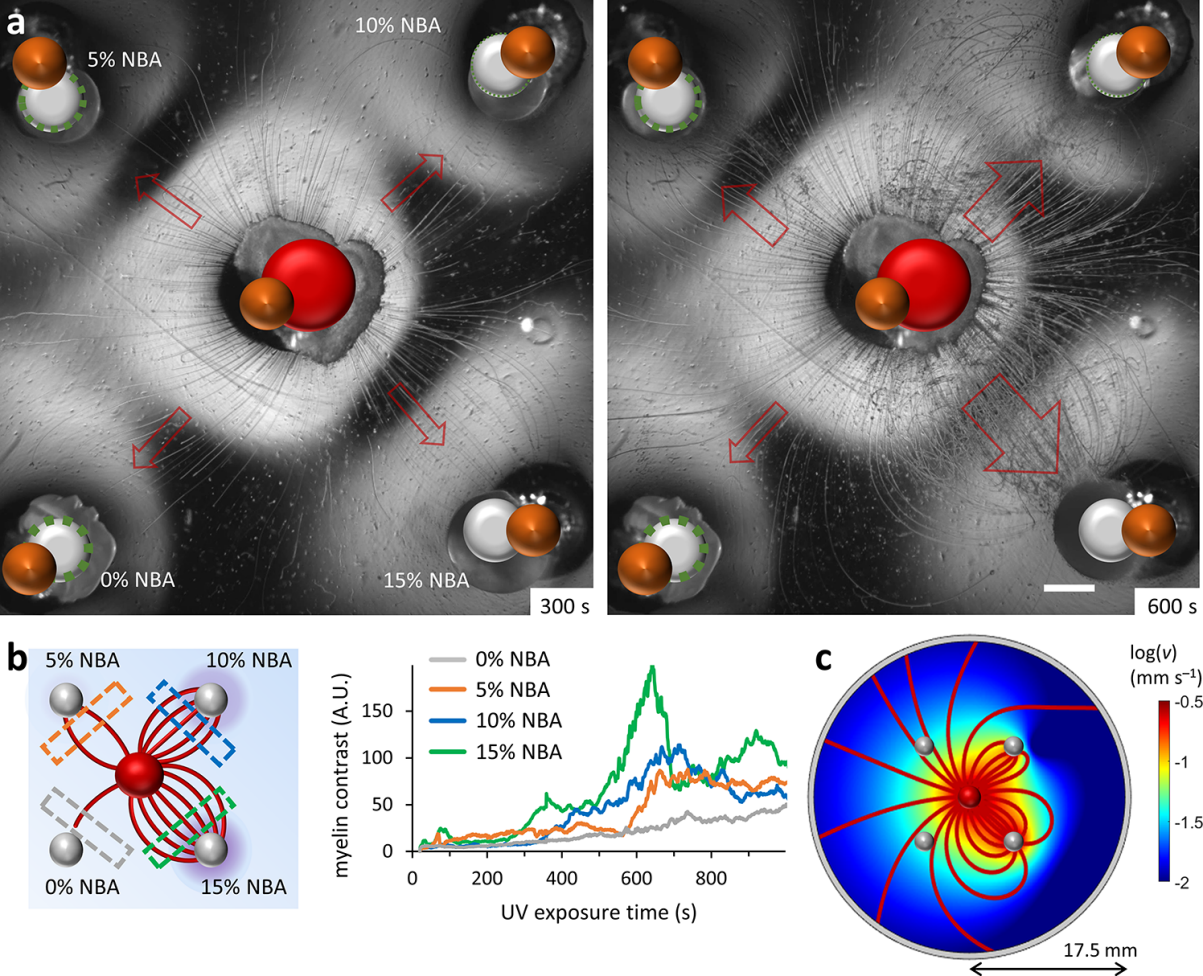
(a) Competition for myelins in a system with 4 drain droplets with varying NBA content (0–15 wt %) around a C_12_E_3_ source droplet in the center (UV is turned on at *t* = 0 s). The scale bar represents 1 mm. (b) The optical contrast of the myelins in the dashed boxes close to the drain droplets (as shown in the scheme left) is followed via image analysis to present the asymmetry in myelin attraction to the drains with different NBA contents over time. (c) Simulated flow velocity profiles among the source and drains with different strengths, corresponding to the drains with 0–15 wt % NBA, as positioned in the experiment in panel (a).

We explore how the photoactive drains allow for selective connections in settings where multiple drains compete for myelins. First, we place a C_12_E_3_ source at equidistance between four simultaneously photoactivated drains, with NBA contents varying from 0 to 5, 10, and 15 wt % (Figure 3a and Movie 3). Upon UV exposure of the entire system, the myelins first curve toward the 10 and 15 wt % NBA drains, later followed by the 5 wt % drain. A limited number of myelins tethers to the 0 wt % drain, albeit without curving, indicating only limited attraction. The time-dependent density of the myelins moving toward the different drains was followed via image analysis, and Figure 3b displays the contrast of the myelins in the proximity of the drains. After 500 s of UV exposure, the asymmetry in myelin attraction is exacerbated as the density of myelins moving toward the 15 wt % drain suddenly increases. In the following minutes, the same phenomenon occurs at the other photoactivated drains, albeit at lower intensity. The patterns of the myelins as they curve toward the different drains are comparable to the flow profiles that are predicted by the kinetic model (Figure 3c) when using depletion rates (i.e., values of *k*_2_) that match with the experimental observed surface tension dynamics for the 0, 5, 10, and 15 wt % NBA drains in Figure 2b. When 15 wt % NBA gets saturated with C_12_E_3_ at approximately 600 s, more filaments move toward the 10 wt % drain and, subsequently, to the 5 wt % drain. The final attraction of the filaments to the 0 wt % drain can be rationalized by the Marangoni flow transferring acid that leaked from the NBA-loaded drains toward this initially NBA-free drain. Gratifyingly, when four equal drains with 15 wt % NBA are positioned around the source, a symmetric distribution of myelins growing in an equal extent toward all drains was observed (Figure S5 and Movie 3).

**Figure 4:**
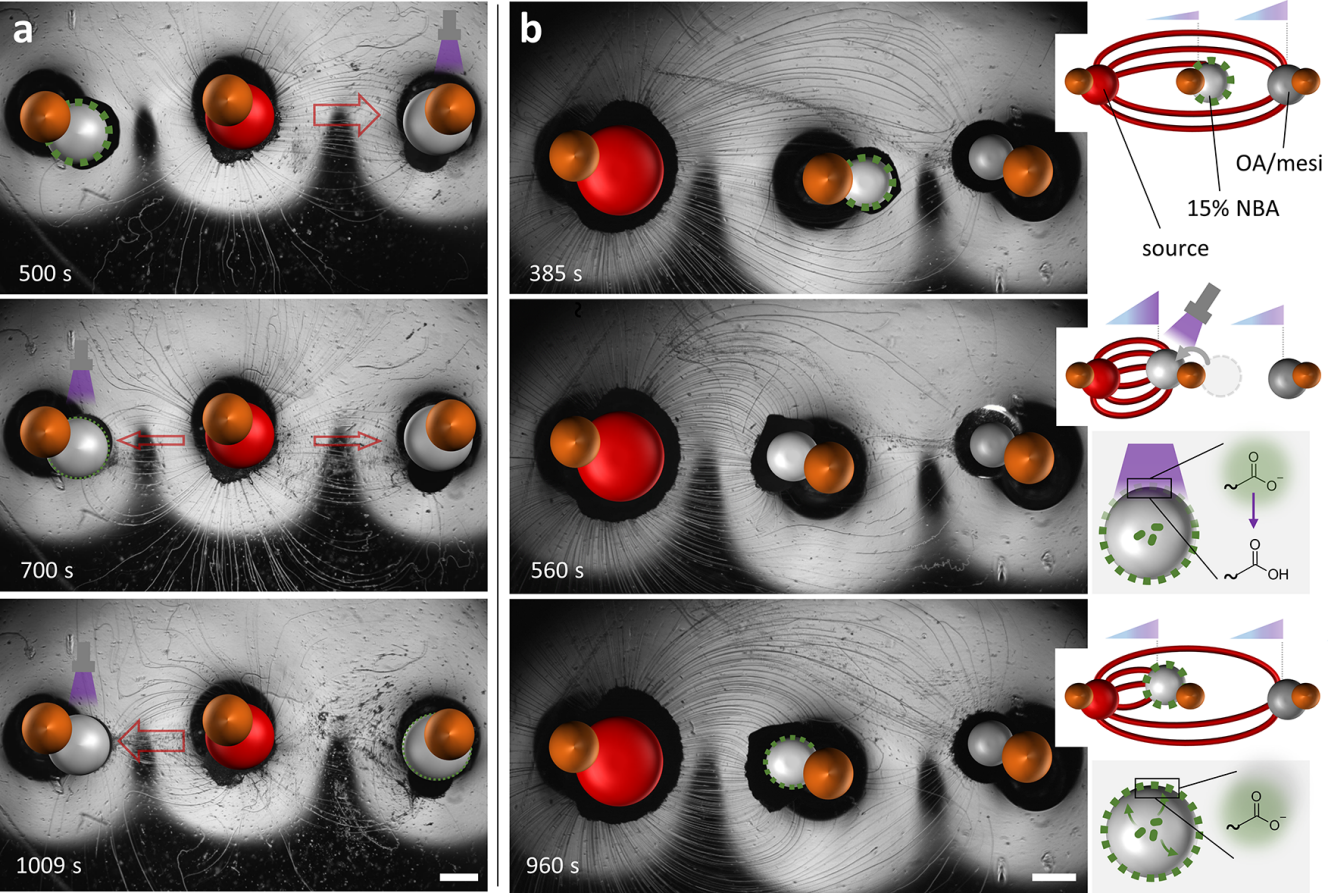
Trajectory of myelin filaments among photoactive drains upon transient UV exposure. (a) Optical microscopy images of a source droplet placed at equidistance in between two 15 wt % NBA drains. From *t* = 15 s, the right drain is exposed to UV, while from *t* = 510 s, the left drain is exposed, inducing a change in myelin trajectory as indicated by the red arrows. (b) Optical microscopy images of a source droplet (left), a 15 wt % NBA drain (middle), and a 1:1 OA/mesi drain (right). The system is exposed to UV in the time frame *t* = 431–610 s. At *t* = 385 s, the myelins are attracted toward the OA/mesi drain, and from *t* = 560 s, they redirect toward the photoactivated NBA drain. When UV is turned off, a fraction of the myelin redirects back to the OA/mesi drain (960 s). The inset schemes show the hypothesized reformation of the liquid crystalline coating: Transient UV exposure leads to NaO protonation and partial disintegration of the coating at the top of the drain droplet. When UV is turned off, repartition of NaO to the droplet interface helps forming the coating anew. The scale bars represent 1 mm.

Second, we place a C_12_E_3_ source droplet between two equidistant 15 wt % NBA drains, which are subsequently exposed to UV with a focused beam (Figure 4a and Movie 4)**.** The myelins rapidly migrate toward the UV-exposed drain, while myelin attraction toward the other, non-irradiated drain is minimal. After approximately 500 s, the other drain is exposed to UV, and myelins get attracted toward this second drain as well. In the meantime, myelins are still attracted toward the first drain, indicating that this drain is still active, even though it is no longer exposed to UV. Only at *t* = 1005 s, all myelins rapidly redirect toward the second, UV-exposed drain. During the third period, a short UV exposure of drain 1 (70 s) redirects some of the myelins from drain 2 to drain 1 again. This sequence is repeated in the following time periods, with the myelins redirecting back and forth between the two drains.

Third, to further understand the behavior of NBA drains under transient UV exposure, we assessed a setting where an NBA drain, which attracts myelins upon photoactivation, competes with an OA/mesi drain (without NaO), which is continuously active. We place a 15 wt % NBA drain in between a source droplet and an OA/mesi drain, as shown in Figure 4b (Movie 5). First, the myelins are attracted by the OA/mesi drain, despite its larger distance from the source, and curve around the NBA drain. Upon exposing the NBA drain to UV, the myelins redirect within 30 s their trajectory toward the photoactivated drain. This indicates that small changes in the UV-triggered surface tension gradient can have a large impact on the myelin trajectory. Comparable behavior was observed for OA/mesi and NBA drains placed at equidistant positions from the source (Figure S6 and Movie 5). As the UV exposure continues for 180 s, the attraction between the myelins and the NBA drain increases to a point where the drain rotates around the steel pillar to get closer to the source droplet and, subsequently, the majority of the myelins are directed toward this drain. Intriguingly, turning off UV leads to another change in the myelin trajectory: a fraction of the myelins disconnects from the NBA drain and curves back toward the OA/mesi drain. These observations imply a decrease in surface tension gradient around the NBA drain when the photoproduction of acid stops.

The decline in myelin attraction during the UV-off periods can be attributed to the role of the liquid crystalline coating in regulating the ability of the drain to absorb surfactant molecules from the a/w interface. Photoinduced degradation of the coating upon NaO protonation is anticipated to happen primarily at the interface, as the optical density of the liquid crystalline coating shields NBA in the core of the drain from being photoactivated. Next, when the UV is (temporarily) turned off, we envision that the high concentration of NaO (10 wt %) present in the drain interior acts as a reservoir and is the main factor in (partly) restoring the liquid crystalline coating of the droplet surrounded by a weak buffer solution (0.03 M sodium alginate). Together, these results imply that time-variant myelin attraction patterns toward different drains can be designed by employing the dependency of the drains on NaO concentration, UV exposure, and C_12_E_3_ accumulation.

### 2.4 Transfer of Cargo by Myelins

**Figure 5:**
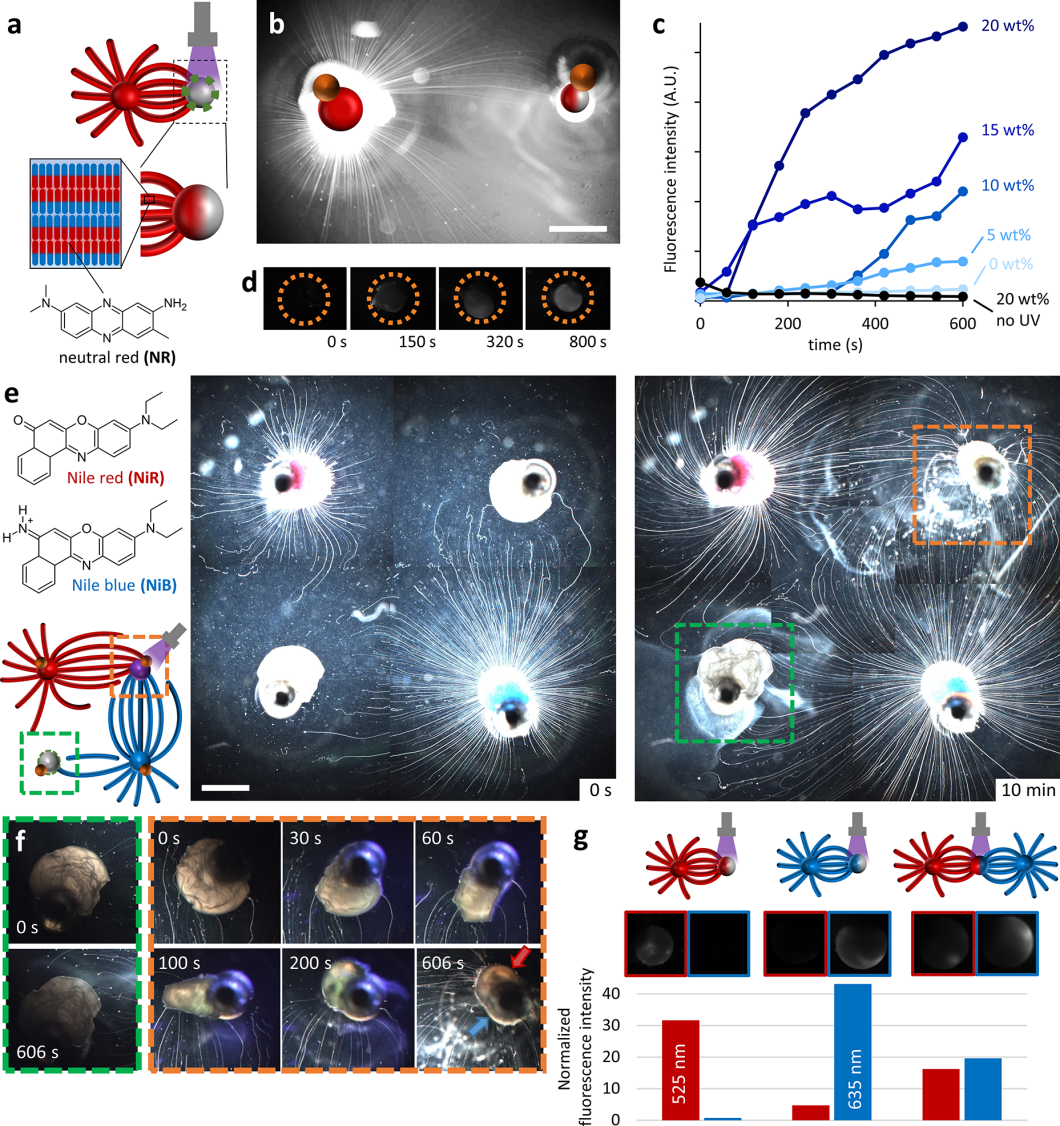
Photocontrolled transfer of fluorescent cargo dyes by the myelins. (a) A source droplet loaded with neutral red (3 wt % NR) grows myelins that transfer their NR cargo to the 20 wt % NBA drain. (b) Fluorescence microscopy image with enhanced brightness to highlight the NR loading in the myelins. (c) Time-dependent fluorescence intensity of drain droplets containing 0–20 wt % NBA in the presence of myelins loaded with NR. UV is exposed in cycles of [60 s UV on + 20 s UV off]. (d) Consecutive fluorescence microscopy images corresponding to the experiment in panel (c) with the 15 wt % NBA drain droplet. (e) Optical microscopy images of a system with two sources with 1 wt % Nile red (NiR) and 1 wt % Nile blue (NiB) and two 10 wt % NBA drain droplets. The top right drain is exposed to UV for 10 min. (f) Zoom-in on the drains reveals the non-UV-exposed drain (green box) to remain unchanged; the UV-exposed drain (orange box) shows a transition from a liquid crystalline structure to a spherical oil droplet that also shows red and blue hues as indicated by the arrows. (g) Fluorescence of 15 wt % NBA drains after 5 min UV exposure in the presence of a source droplet loaded with NiR (1 wt %, left), a source droplet loaded with NiB (1 wt %, middle), and two source droplets loaded with NiR and NiB (1 wt %, right). The fluorescence intensity is normalized to the fluorescence intensity of the respective drain droplet at *t* = 0 s. The scale bars represent 1 mm (b) and 2 mm (e), respectively.

Control over the myelin organization opens the possibility to use them for transport of small organic compounds that are dispersed in the bilayers of the C_12_E_3_-based lamellar phase.^62^ In this context, we envision that the delivery of cargos to the core of the drain can be regulated by the liquid crystalline coating (Figure 5a). We load C_12_E_3_ source droplets with 3 wt % neutral red (NR), which provides the myelins with a fluorescent signal—albeit with lower intensity compared to the much larger source droplet (Figure 5b). A negligible amount of NR leaks out to the surrounding aqueous solution (Figure S7). To assess whether photoactivation of the drains can be used to control the delivery of NR by the myelins, we compare the time-dependent fluorescence intensity of two 20 wt % NBA drains upon exposure to UV and in the absence of UV (Figure 5c). The photoactivated drain is exposed to UV in cycles of [60 s on +20 s off] such that the fluorescence intensity can be probed via fluorescence microscopy during the UV-off periods. The fluorescence intensity of the non-UV exposed drain remains 0 despite being present for 14 min at the a/w interface in the presence of NR-loaded myelins. The photoactivated drain however acquires an increasing fluorescence intensity over time: a sharp rise occurs after the second UV-on cycle (120 s of cumulated UV exposure) when the onset of the liquid crystalline coating disintegration is expected. The fluorescence increases in the subsequent cycles and then levels off, presumably as saturation of the drain by C_12_E_3_ declines attraction of new myelins with NR. For a 15 wt % NBA drain, we observe a comparable evolution of the fluorescence intensity upon UV exposure: Fluorescence microscopy images show the progression of NR within the drain, starting from the side that faces the source droplet until, eventually, the entire droplet is fluorescent (Figure 5d). Further reducing the NBA concentration in the drain suppresses the uptake of NR significantly, and a drain without NBA remains nonfluorescent upon UV exposure.

To deliver signals from different sources that are loaded with fluorescent cargos that can be distinguished from each other, we combined Nile red (NiR) with the comparable benzophenoxazine dye Nile blue (NiB). Two sources with 1 wt % NiR and 1 wt % NiB were deposited together with two 10 wt % NBA drains in a square, as shown in Figure 5e. Upon localized exposure to UV, the drain in the top right attracts myelins from both sources at a fast rate, whereas the progression of myelins in the direction of the non-UV-exposed drain is much slower (Movie 6). Even though the conversion of the UV-exposed drain from the liquid crystalline to liquid state suggests the delivery of the dye cargos to the drain interior, the red and blue colors are only weakly visible in optical microscopy (Figure 5f). For a comparable experiment conducted in fluorescence microscopy, however, the NiR-loaded source (1 wt %) gives a stronger signal with an excitation wavelength of 525 nm, and the NiB-loaded source (1 wt %) with 635 nm. When exposing an NBA drain to UV, attraction of NiB-loaded myelins results in a strong fluorescence of the drain at 635 nm, while the fluorescence at 525 nm shows only a minor increase (Figure 5g). *Vice versa*, the same experiment with NiR-loaded myelins results in a drain that displays only a strong increase in fluorescence at 525 nm. Gratifyingly, UV exposure of an NBA drain in the presence of both a NiR-loaded source and NiB-loaded source results in an increase in fluorescence at 525 nm as well as 635 nm. Together, these findings demonstrate that C_12_E_3_ myelins are reliable vehicles for the transport of molecular cargo, and the use of photoactivated drains allows to establish delivery points at the a/w interface.

## 3 Conclusions

We established chemical communication via myelin filaments of which the trajectory from source to drain droplets can be directed upon photocontrol. Using droplets as elementary units allows to encompass the compounds that enable sending or receiving chemical signals in individually targetable nodes. By UV-triggering floating droplets to deplete the surfactant from the air–water interface upon photochemical disintegration of their liquid crystalline coating, localized surface tension gradients can be generated such that myelins are attracted and deliver their chemical signals. We envision that our approach—based on molecular building blocks that are simple, easily accessible, and amenable to chemical modification—provides a transferable design principle to spatiotemporally controlled droplet networks.

To arrive at next generations of “smart” matter, effectively coordinated self-organization benefits from connections that “guide” molecular inputs along adaptive, interactive pathways among sender and receiver agents. In this regard, we note that a fully reconfigurable network requires control over the lifetime of the myelins—now in the order of hours (Figure S8)—upon controlled disassembly, for example, by chemical disintegration of the amphiphile. Swarms of agents that exchange molecular information open potential in physicochemical systems that not only compute based on chemical input but concomitantly form functional structures, for example, in self-organizing devices that “determine” the path for a sample along a cascade of analyses in lab-on-a-chip or droplet-handling platforms^63^ or in associative memories that are encoded by the spatial organization of the network and operate in analogy to network-shaped memories as recently reported for *P. polycephalum*.^64^

## Supporting Information Available

Optical microscopy recording of a 20 wt% NBA drain droplet (1.0 μL) deposited at an aqueous solution (0.63 wt % sodium alginate and 100 μM C_12_E_3_) (AVI)
Optical microscopy recordings of a C_12_E_3_ source droplet (2.0 μL) and drain droplet (2.0 μL and 10–20 wt % NBA) positioned at steel pillars (AVI)
Optical microscopy recordings of competition for myelins in a system with 4 drain droplets (2.0 μL) around a C_12_E_3_ source droplet (2.0 μL) in the center (MP4)
Optical microscopy recording of a C_12_E_3_ source droplet (1.0 μL) placed at equidistance in between two 15 wt % NBA drains (2.0 μL) (MP4)
Optical microscopy recordings of a C_12_E_3_ source droplet (left movie, 2.0 μL; right movie, 1.0 μL), a 1:1 OA/mesi drain (2.0 μL), and a 15 wt % NBA drain (2.0 μL) placed in different spatial conformations for two different experiments (MP4)
Optical microscopy recording of two C_12_E_3_ source droplets (1.0 μL) with Nile red (1 wt %, top left) and Nile blue (1 wt %, bottom right) and two 10 wt % NBA drain droplets (2.0 μL) (MP4)
Additional experimental details, materials and methods, details on the model and the simulations, and supporting figures (PDF)

## Supplementary Material

ja3c12882_si_001.avi

ja3c12882_si_002.avi
