# Supplementary material for: Reconfigurable Droplet–Droplet Communication Mediated by Photochemical Marangoni Flows
Source: J Am Chem Soc. 2024 Feb 23;146(9):6006–15. doi: 10.1021/jacs.3c12882 (PMC10921405; doi:10.1021/jacs.3c12882)
Supplement: Supplementary file 7 — ja3c12882_si_007.pdf [file ja3c12882_si_007.pdf]

Reconfigurable droplet-droplet communication mediated by photo-chemical Marangoni flows.

*Anne-Déborah C. Nguindjel, Stan C. M. Franssen & Peter A. Korevaar**

Institute for Molecules and Materials, Radboud University, Heyendaalseweg 135, 6525 AJ Nijmegen, The Netherlands E-mail: p.korevaar@science.ru.nl

Supplementary Information

Table of contents:

Materials and methods 2

Details on the model and the simulations 4

Supplementary Figures 1 – 9 8

Description of Movies 1 – 6 15

References 15

Materials and methods.

Sodium oleate (95 %) was purchased from ABCR GmbH & KG, oleic acid (99.0 %) from Sigma-Aldrich and Fluorochem Ltd, sodium alginate (99.0 %) from Sigma-Aldrich, sodium chloride (99.0 %) from VWR Chemicals, 2-nitrobenzaldehyde (≥ 99.0 %) from Merck, mesitylene (98.8 %) from Aldrich, tri(ethylene glycol) monododecyl ether (≥ 95 %) from TCI Ltd, neutral red (≥ 90 %) from Janssen, Nile blue A (≥ 75 %) and Nile red (≥ 97.0 %) from Sigma-Aldrich. All materials were used as received.

UV Lamps: Unless specified otherwise, a Tank007 UV lamp (365 nm LED, 75 mW/cm^2, beam diameter 3 mm) was used to irradiate individual drain droplets at the interface. A Kessil PR160L UV lamp (370 nm LED, 130 mW/cm^2, beam diameter 6 cm) was used to irradiate the entire a/w interface (Fig. 3 and Fig. S5). The intensity of both lamps at 365 nm was measured with a Thorlabs PM100A power meter. The distance between the lamp and the sensor was set at 4 cm, similar to the distance between the lamp and samples during experiments.

Drain solutions: To prepare the drain solutions, we used a 1:1 v/v OA/mesi mixture, with varying NaO and NBA weight percentages as indicated. Unless specified otherwise, the drain solutions contained 10 wt% NaO. NBA and NaO were dissolved in the OA/mesi mixture upon sonication.

Aqueous bulk solution: An aqueous solution of 17 mM sodium chloride and 0.63 wt% sodium alginate is systematically used as aqueous bulk solution.

Self-organization experiments: Devices made of steel pillars were used to immobilize buoyant droplets at specific locations of the a/w interface (Fig. S8). The concave meniscus formed by the pillars induces the 'Cheerios' effect, which attracts the droplets and keeps them in place.[1] The devices were assembled by heating steel rods (10 mm height, diameter 1 mm) at the base with a torch flame and fusing them to a Plexiglas plate (thickness 2 mm). Once cooled, the device was placed in a Petri dish (lid of a Falcon 35 mm dish, diameter 38 mm, height 4.5 mm, used as received) filled with the aqueous bulk solution. Prior to the deposition of the source droplet, the surface tension of the sodium alginate solution was decreased to approx. 28 mN m^{-1} by adding a trace amount of amphiphile solution to the interface: this avoids the source droplet to be exposed to large surface tension gradients that rapidly tear apart the droplet upon deposition.[2] The droplets were deposited at the water-pillar contact point with a Gilson pipette, starting with the $C_{12}E_3$ source droplets, followed by the drain droplets. All experiments were performed at room temperature.

IR measurements: A Shimadzu spirit-T IR-ATR was used to measure the IR spectra of the different drain solutions (2 µL sample). Calibration curves are obtained by plotting the transmittance at 1345 and 1531 cm^{-1} of drain solutions containing respectively 0; 5; 10; 15 and 20 wt% NBA (1:1 v/v OA/mesi with 10 wt% NaO). Using the calibration curves, the evolution of the NBA content in drain solutions exposed to UV was determined by monitoring the transmittance at 1345 and 1531 cm^{-1} of 15 wt% NBA solutions exposed to UV for increasing time periods (0 to 420 s).

UV-Vis spectroscopy: All measurements are performed with a Tecan Spark plate reader, using 384 transparent well plates. For pure drain solutions, a volume of 80 µL was used; to study the formation of the liquid crystalline phase, 50 µL of drain solution was added to 30 µL of aqueous bulk solution.

Testing leakage of dyes from myelins: In a Petri dish filled with 5 mL MQ water, a source droplet (2 µL) loaded with the respective dye was deposited at the a/w interface. 150 µL aliquots were extracted from the bulk solution with an interval of 3 minutes, and their fluorescence intensity was measured in a Tecan plate reader (λ_{ex} = 525 nm; λ_{em} = 565 nm for Neutral red, Nile red and Nile blue dyes). Via a calibration curve, the concentration of the dye in solution was determined.

Surface tension measurements: The dynamic surface tension was measured using a Biolin Scientific Sigma 701 tensiometer in combination with a platinum Wilhelmy plate (19.62 mm x 10 mm). Prior to

each measurement, the Pt plate was cleaned with a flame until red hot, and the balance was equilibrated in air. For each experiment, 3.5 mL of aqueous bulk solution was added in a polystyrene petri dish (35 mm diameter, solution height approx. 3 mm), and the Wilhelmy plate was dipped at the air-water interface, such that the water wetted the plate and formed a meniscus.

Optical microscopy: All optical microscopy images and movies were acquired with an Olympus IX73 dark-field inverted microscope equipped with a Point Grey Grasshopper3 camera. A x1.25 objective was used; brightness and condenser filters were adjusted appropriately, and the videos are recorded at 1 frame per second unless stated otherwise.

ImageJ was used to enhance the visibility of the myelin filaments in Figure S3, by first subtracting the first frame and then auto adjusting the brightness and contrast. To follow the density of myelins attracted towards the different drains in Fig. 3 and Fig. S5, the contrast of the frames was enhanced by subtracting the frame before UV is turned on, and then auto adjusting the brightness and contrast. Next, the contrast of the myelins was measured by imageJ in the rectangles close to the drain, as schematically shown in Fig. 3b and Fig. S5b.

Fluorescence microscopy: An Olympus IX83 inverted fluorescence microscope equipped with a Hamamatsu ORCA Flash 4.0 camera was used to acquire the fluorescence images. The exposure time was set to 500 ms and the samples were excited with an LED lamp at 550 nm, set at 80 % of its intensity and used in conjunction of an U-FYW filter cube. A x2 PLAPON objective was used and the videos are recorded at 1 frame per second unless stated otherwise.

The transfer of neutral red from source to drain is assessed by depositing a 1 μL $C_{12}E_3$ source droplet containing 3 wt% neutral red and 1 μL of drain droplets containing various percentages of NBA on pillars (d = 7 mm), at the air-water interface. The drain droplets are irradiated with UV in 10 consecutive cycles of [60 s ON – 20 s OFF] using a Tank007 UV lamp. The time-dependent fluorescence intensity of the drains is measured in *imageJ* on frames that were acquired when the UV was turned off.

The transfer of Nile red and Nile blue from source to drain is assessed by depositing a 1 μL $C_{12}E_3$ source droplet containing respectively 1 wt% Nile red or Nile blue, and 1 μL of drain droplets containing specified concentrations of NBA and NaO on pillars (d = 7 mm). The a/w interface is exposed to UV with a Kessil 370 lamp, and the fluorescence intensity of the drain is measured in *imageJ* on frames that were acquired when the UV was turned off.

Details on the model and the simulations.

The kinetic model that describes the Marangoni flow profiles from the source to the drain droplets along the a/w interface was reported earlier by our research group in ref. 3, and modified here to describe the photo-active drains and the $C_{12}E_3$ surfactant system. To describe the relationship between amphiphile concentration in the aqueous medium A_m (in mol cm^{-3}), the surface concentration of amphiphiles at the air-water interface Γ (in mol cm^{-2}) and the surface tension γ for $C_{12}E_3$ (in mN m^{-1}), we used the relation:

(1) $\Gamma = \frac{A_m \cdot \Gamma_\infty}{K_3 + A_m}$,

and the Frumkin isotherm[4]:

(2) $\gamma = \gamma_0 + 10^7 \cdot \Gamma_\infty RT \left(ln\left(1 - \frac{\Gamma}{\Gamma_\infty}\right) - \frac{K}{2}\left(\frac{\Gamma}{\Gamma_\infty}\right)^2 \right)$.

Here, γ_0 represents the surface tension of the air-water interface (72 mN m^{-1} for pure water); R the gas constant, T the temperature (T = 293 K), K the adsorption cooperativity (K = 6.2 for $C_{12}E_3$)[4] and Γ_∞ the maximum surface concentration ($\Gamma_\infty = 3.9 \cdot 10^{-10}$ mol cm^{-2} for $C_{12}E_3$).[4]

With $K_3 = 5.3 \cdot 10^{-9}$ mol cm^{-3}, eqs. (1) and (2) predict a surface tension of 28 mN m^{-1} at the cmc of $C_{12}E_3$ ($A_m = 4.5 \cdot 10^{-8}$ mol cm^{-3}).[4] Furthermore, eq. (1) predicts for a saturated air-water interface (*i.e.*, at the cmc) $\Gamma_{CMC} = 3.49 \cdot 10^{-10}$ mol cm^{-2}.

The simulations in Figure 2e were performed by solving the system of differential equations (3–6) using Matlab (R2019a, *ode15s solver*):

(3) $\frac{dA_s}{dt} = -k_1 \cdot A_s \cdot \theta$;

(4) $\frac{d\theta}{dt} = -k_1 \cdot A_s \cdot \theta + k_3 \cdot \Gamma - k_{-3} \cdot A_m \cdot \theta + k_2 \cdot \Gamma$;

(5) $\frac{d\Gamma}{dt} = k_1 \cdot A_s \cdot \theta - k_3 \cdot \Gamma + k_{-3} \cdot A_m \cdot \theta - k_2 \cdot \Gamma$;

(6) $\frac{dA_m}{dt} = \alpha \cdot k_3 \cdot \Gamma - \alpha \cdot k_{-3} \cdot A_m \cdot \theta$.

Here, A_s (in mol cm^{-2}) is the concentration of amphiphiles in the source droplet and filaments, defined as if they are homogeneously distributed over the air-water interface. (Later, we will use the depletion rate of amphiphiles – predicted by the kinetic model – to simulate the flow patterns between source and drain droplets with defined locations at the air-water interface). θ (in mol cm^{-2}) is the surface concentration of the vacant sites at the interface. α represents the ratio of air-water interface per aqueous phase volume (in cm^2/cm^3). k_1, k_2, k_3 and k_{-3} represent the rate constants as shown in Figure 2d, with $K_3 = k_3/k_{-3}$.

When we simulate the uptake of surfactant from the interface of a 100 µM $C_{12}E_3$ solution (Fig. 2e), at t = 0, the amphiphile concentration in the source droplet A_s = 0, since there is no amphiphile source

droplet present in the simulation. We assume here that the amphiphile concentration in the aqueous solution A_m is constant and, due to a large excess of $C_{12}E_3$ (0.1 mM; $A_m = 1.0 \cdot 10^{-7}$ mol cm^{-3}), does not change upon depletion of $C_{12}E_3$ from the air-water interface by a drain droplet that is deposited at $t = 0$ s. Furthermore, $\Gamma(t = 0) = \frac{A_m \cdot \Gamma_{CMC}}{K_3 + A_m}$ and $\theta(t = 0) = \Gamma_{CMC} - \Gamma(t = 0)$, as elaborated in ref 3.

The simulations are performed with $k_1 = 1 \cdot 10^8$ cm^2 mol^{-1} s^{-1}; $k_3 = 3 \cdot 10^{-3}$ s^{-1} and $\alpha = 2$ cm^2/cm^3. The value of k_3 is chosen such that the model predicts a re-equilibration of the rise in surface tension $\Delta\gamma$ upon application of the drain over a time course of approx. 40 seconds, in agreement with the experimental observations in Figure 2b. The value of α corresponds to the surface/volume ratio for a solution with a height of 0.5 cm.

The rate constant that characterizes the activity of the drain (k_2) was varied from $k_2 = 0.01 \cdot k_3$ to $k_2 = 0.5 \cdot k_3$ in Figure 2e, where we simulate the uptake of $C_{12}E_3$ from the a/w interface of an aqueous solution by the drain.

Next, to simulate the Marangoni flow profiles amongst source and drain droplets at an a/w interface, we include a 1 µL $C_{12}E_3$ source droplet in the system, which implies $A_s(t = 0) = 2.84 \cdot 10^{-7}$ mol cm^{-2}. Via the rate equations (eqs. 3 – 6) we simulate the depletion rate of the amphiphile towards the aqueous phase Φ_{water} (in mol cm^{-2} s^{-1}) by:

(7) $\Phi_{water} = k_3 \cdot \Gamma - k_{-3} \cdot A_m \cdot \theta$;

and the depletion rate towards the drain Φ_{drain} (in mol cm^{-2} s^{-1}) via:

(8) $\Phi_{drain} = k_2 \cdot \Gamma$.

At the core of our approach to describe the Marangoni flow pattern from source to drain is the assumption that the depletion of amphiphile from the air-water predominantly occurs at 1) the boundary of the Petri dish, where a downward flow emerges that transfers amphiphiles from the a/w interface to the underlying solution, and 2) at the drain. Next, we calculate $v_{source}(R)$ at the edge of the flow zone with radius R (*i.e.* close to the Petri dish boundary) via $\Phi_{water} \cdot a = \Gamma \cdot a_{ring}$, with a the total area of the air-water interface ($a = 9.6$ cm^2). Assuming that $v_{source}(R) \cdot (1\text{ s}) << R$, $a_{ring} = 2\pi R \cdot v_{source}(R) \cdot (1\text{ s})$, which yields:

(9) $v_{source}(R) = \frac{\Phi_{water} \cdot a}{2\pi\Gamma}$.

Via the relationship $v \sim r^{-1}$ (as was found by Bandi et al.[5] and we established for our myelin-surfactant system in ref. 3), one can find:

(10) $v_{source}(r) = v_{source}(R) \left(\frac{r}{R}\right)^{-1}$.

In analogy, we define for the flow towards the drain v_{drain}:

(11) $v_{drain}(R_{drain}) = -\frac{\Phi_{drain} \cdot a}{2\pi\Gamma R_{drain}}$;

(12) $v_{\text{drain}}(r_{\text{drain}}) = v_{\text{drain}}(R_{\text{drain}}) \left(\frac{r_{\text{drain}}}{R_{\text{drain}}}\right)^{-1}.$

Here, R_{drain} equals the radius of the drain. We included a (–) sign in the expression for v_{drain} (eq. 11), since the flow velocity is oriented towards the drain, whereas v_{source} is directed away from the source.

For every (x,y) coordinate, we calculate r via $r = \sqrt{(x - x_{\text{source}})^2 + (y - y_{\text{source}})^2}$, and r_{drain} via $r_{\text{drain}} = \sqrt{(x - x_{\text{drain}})^2 + (y - y_{\text{drain}})^2}$, where x_{source} and y_{source} represent the x- and y-positions of the source, and x_{drain} and y_{drain} the x- and y-positions of the drain, respectively.

Next, the x- and y-components of v_{source} at (x,y) are found via:

(13) $v_{\text{source,x}}(x, y) = \frac{v_{\text{source}}(r)\cdot(x - x_{\text{source}})}{\sqrt{(x - x_{\text{source}})^2 + (y - y_{\text{source}})^2}}$;

(14) $v_{\text{source,y}}(x, y) = \frac{v_{\text{source}}(r)\cdot(y - y_{\text{source}})}{\sqrt{(x - x_{\text{source}})^2 + (y - y_{\text{source}})^2}}$;

and the x- and y-components of v_{drain} at (x,y) via:

(15) $v_{\text{drain,x}}(x, y) = \frac{v_{\text{drain}}(r_{\text{drain}})\cdot(x - x_{\text{drain}})}{\sqrt{(x - x_{\text{drain}})^2 + (y - y_{\text{drain}})^2}}$;

(16) $v_{\text{drain,y}}(x, y) = \frac{v_{\text{drain}}(r_{\text{drain}})\cdot(y - y_{\text{drain}})}{\sqrt{(x - x_{\text{drain}})^2 + (y - y_{\text{drain}})^2}}$.

Then, the x-component of the velocity $v_{\text{x}}(x,y)$ equals $v_{\text{source,x}}(x,y)$ + $v_{\text{drain,x}}(x,y)$, and the y-component $v_{\text{y}}(x,y)$ equals $v_{\text{source,y}}(x,y)$ + $v_{\text{drain,y}}(x,y)$, such that the total flow velocity $v(x, y) = \sqrt{v_x(x, y)^2 + v_y(x, y)^2}$.

To simulate the flow profile amongst one source and 4 different drains in Figure 3c, we first calculated $v_{\text{drain,1}}(R_{\text{drain}})$ based on the value of Φ_{drain} that was found with $k_2 = 0$ s^{-1} (representing the 0 wt% NBA drain, using eq. 8); $v_{\text{drain,2}}(R_{\text{drain}})$ based on the value of Φ_{drain} that was found with $k_2 = 3\cdot10^{-5}$ s^{-1} (representing the 5 wt% NBA drain); $v_{\text{drain,3}}(R_{\text{drain}})$ based on the value of Φ_{drain} that was found with $k_2 = 6\cdot10^{-4}$ s^{-1} (representing the 10 wt% NBA drain) and $v_{\text{drain,4}}(R_{\text{drain}})$ based on the value of Φ_{drain} that was found with $k_2 = 1.5\cdot10^{-3}$ s^{-1} (representing the 15 wt% NBA drain), respectively. Next, we calculated $v_{\text{source}}(R)$ based on the value of Φ_{water} that was found with $k_2 = (0 + 3\cdot10^{-5} + 6\cdot10^{-4} + 1.5\cdot10^{-3})$ s^{-1} (representing a system with the 0, 5, 10 and 15 wt% NBA drains present, *i.e.* $k_2 = 0$; $k_2 = 0.01\cdot k_3$; $k_2 = 0.2\cdot k_3$; $k_2 = 0.5\cdot k_3$, respectively). In analogy to eqs. 13–16, we calculated the x- and y-components of v_{source} and $v_{\text{drain,1–4}}$, which allows to calculate the x- and y-component of the overall velocity:

(20) $v_{\text{x}}(x, y) = v_{\text{source,x}}(x, y) + \sum_{i=1}^{4} v_{\text{drain,i,x}}(x, y)$,

(20) $v_{\text{y}}(x, y) = v_{\text{source,y}}(x, y) + \sum_{i=1}^{4} v_{\text{drain,i,y}}(x, y)$;

which in turn allows to compute the total magnitude and direction of the velocity for every position (x,y).

The simulations shown in Figures 2f and 3c where performed in Matlab (R2019a). Φ_{water} and Φ_{drain} were simulated via the kinetic model discussed above. Next, we computed the flow velocity and direction at every position (x, y) in a 200x200 matrix that represents a square of 35 by 35 mm^2, with source and drain(s) placed at fixed positions in the matrix. The heat maps represent the flow velocity within a radius of 17.5 mm around the source; the red curves indicate the streamlines (plotted via the *streamline* function in Matlab) from the source towards the drain(s). The simulations are performed with R_{drain} = 0.5 mm and R = 17.5 mm.

The surface tension gradient $\frac{\partial \gamma}{\partial u}$ profiles in Figure S4 are computed based on the velocity profiles in Figure 2f via the relationship $\eta \frac{\partial v}{\partial z} = \frac{\partial \gamma}{\partial u}$. With viscosity η = 5 mPa.s for the NaAlg/water solution[2] and velocity $v = 0$ at z = –1 mm from the air-water interface (as observed in our measurements in ref. 3), $\frac{\partial \gamma}{\partial u}$ in $\left(\frac{\mathrm{mN/m}}{\mathrm{m}}\right)$ equals $5 \cdot v$ (in mm/s).

Supplementary Figures

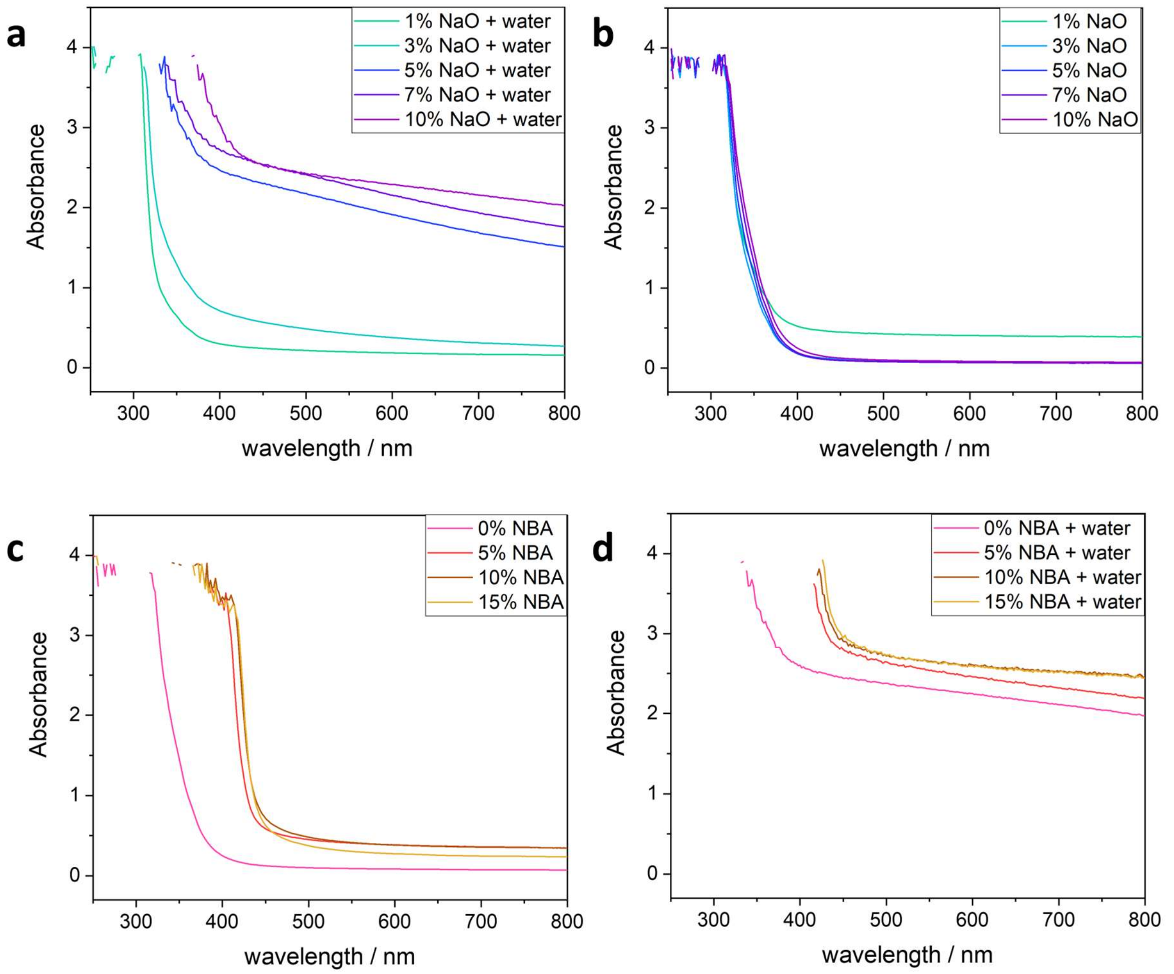

Figure S1. UV-Vis spectra for varying compositions of the drain solution. **a)** Drain solutions with 1:1 mesi/OA and varying NaO wt%, that are mixed with water in a 5:3 volume ratio. **b)** Drain solutions with 1:1 mesi/OA and varying NaO wt% in the absence of water. **c)** Drain solutions with 1:1 mesi/OA, 10 wt% NaO and varying wt% of NBA. **d)** Drain solutions with 1:1 mesi/OA, 10 wt% NaO and varying wt% of NBA, mixed with water in a 5:3 volume ratio.

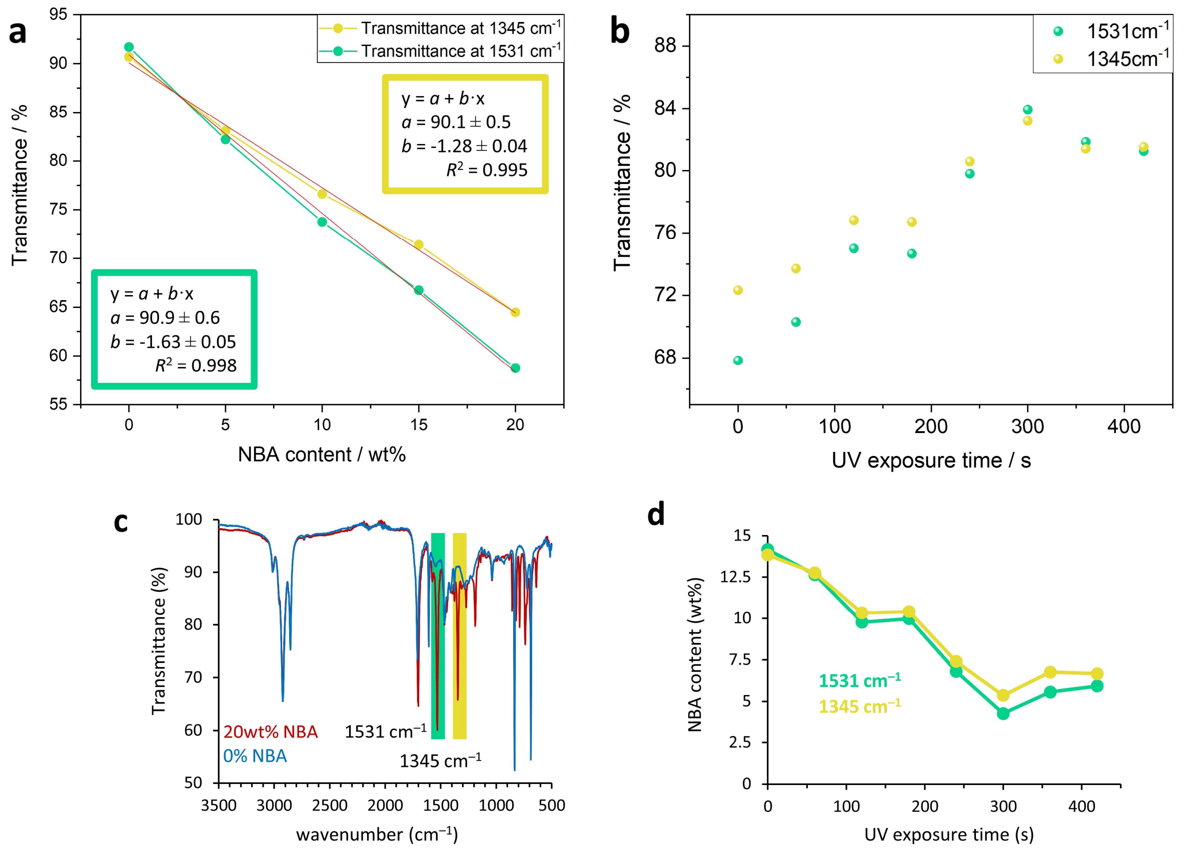

Figure S2. a) NBA content calibration curves obtained by plotting the transmittance at 1345 cm^{-1} (yellow) and 1531 cm^{-1} (green) of drain solutions (1:1 mesi/OA with 10 wt% NaO) containing 0; 5; 10; 15 and 20 wt% NBA, respectively. The inserts show the results of a linear fit through the data. **b)** Transmittance vs. UV exposure time for drain solutions (1:1 mesi/OA with 10 wt% NaO) starting with 15 wt% NBA. **c)** IR-ATR spectra of drain droplets containing 0 and 20 wt% NBA. **d)** NBA content vs. UV exposure time, for drain solutions starting at 15 wt% NBA. The IR transmittance at 1531 and 1345 cm^{-1} was used to determine the NBA concentration.

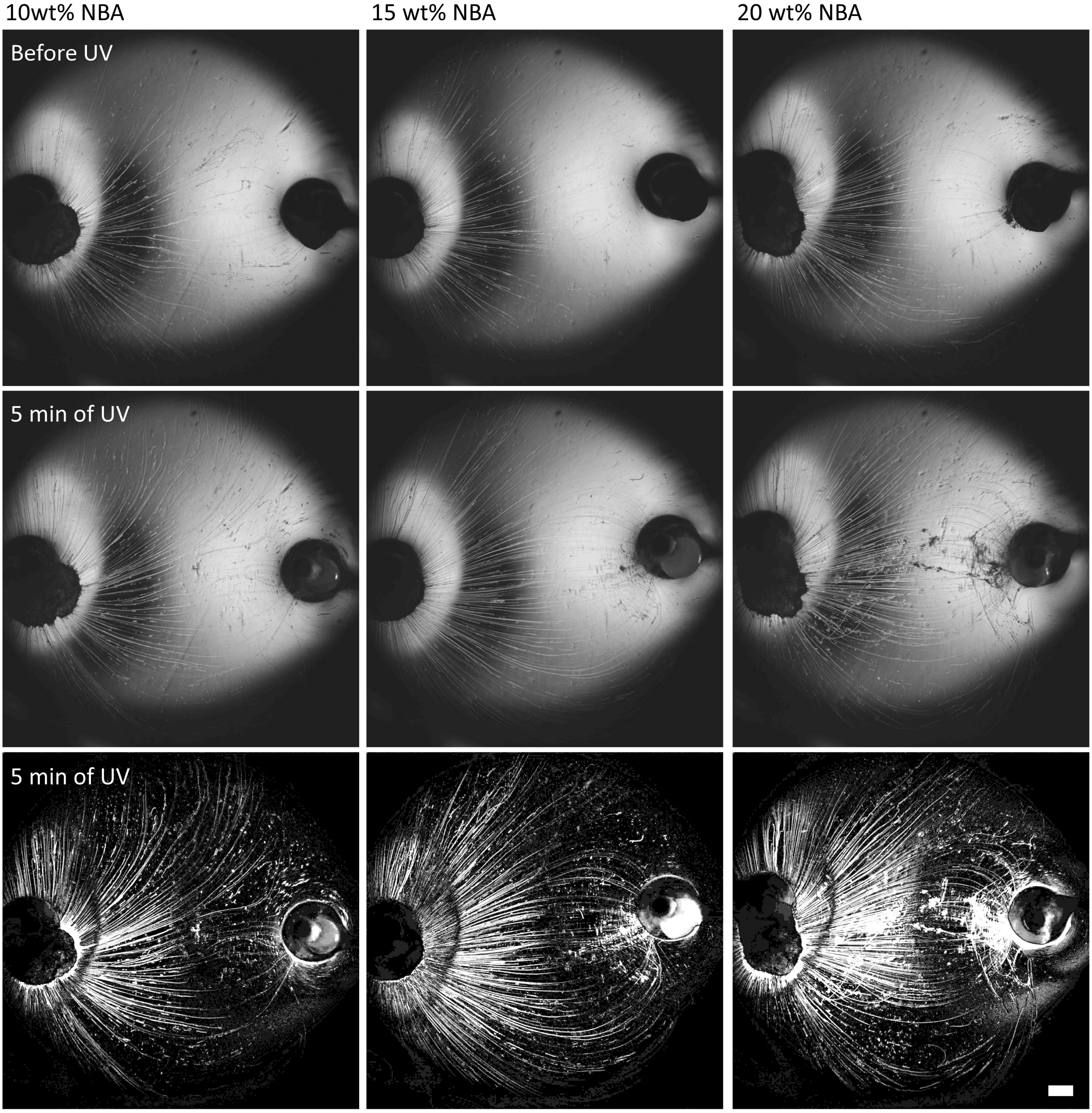

Figure S3. Optical microscopy images of drains with 10 wt% NBA, 15 wt% NBA and 20 wt% NBA, before (top row) and after 5 min of UV exposure (middle row). To highlight the difference in myelin attraction between the different drains, the bottom row shows the frame after 5 min of UV exposure, with the background frame (acquired before deposition of the drain) subtracted and the contrast enhanced in *ImageJ*. The scale bar represents 1 mm.

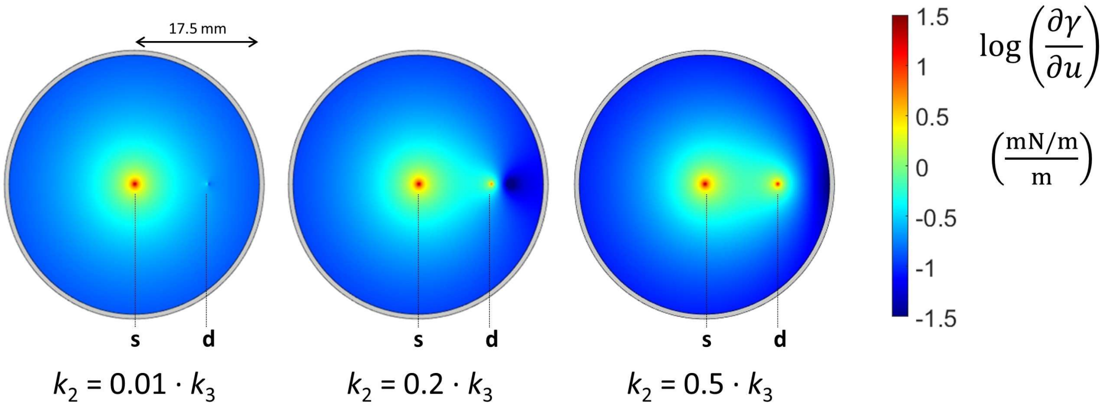

Figure S4. Simulated surface tension gradient $\frac{\partial\gamma}{\partial u}$ profiles among the source (s) and drain (d) droplets, corresponding to the flow velocity profiles in Figure 2f.

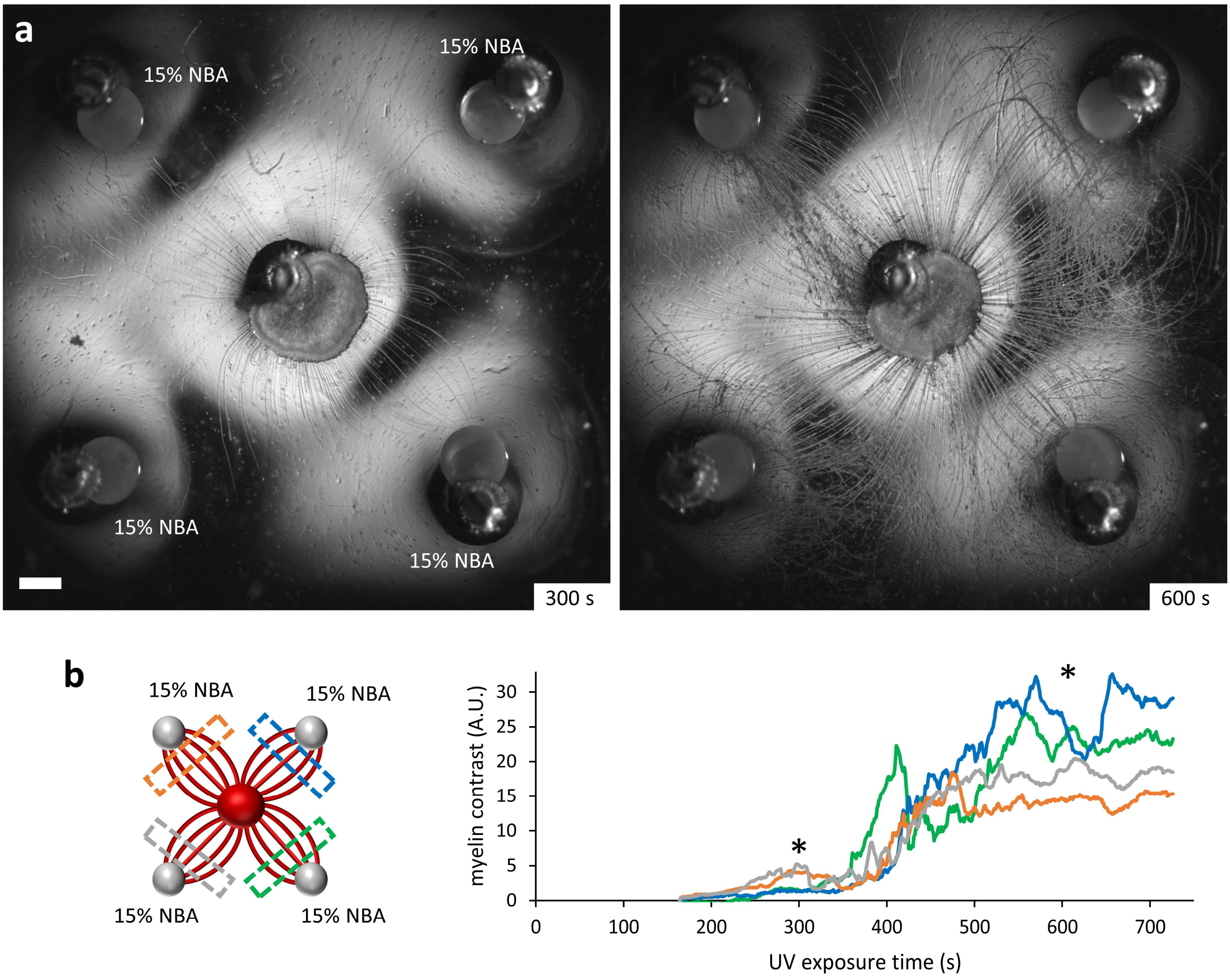

Figure S5. a) Optical microscopy images of myelins in a system with 4 drain droplets with 15 wt% NBA around a $C_{12}E_3$ source in the center (UV is turned on at t = 0 s). The scale bar represents 1 mm. **b)** The contrast of the myelins in the dashed boxes close the drain droplets (as indicated in the scheme left) is followed via image analysis to present the symmetry in myelin attraction towards the 4 drains with 15 wt% NBA over time. The colours in the graph correspond to the colours of the dashed boxes in the scheme left.

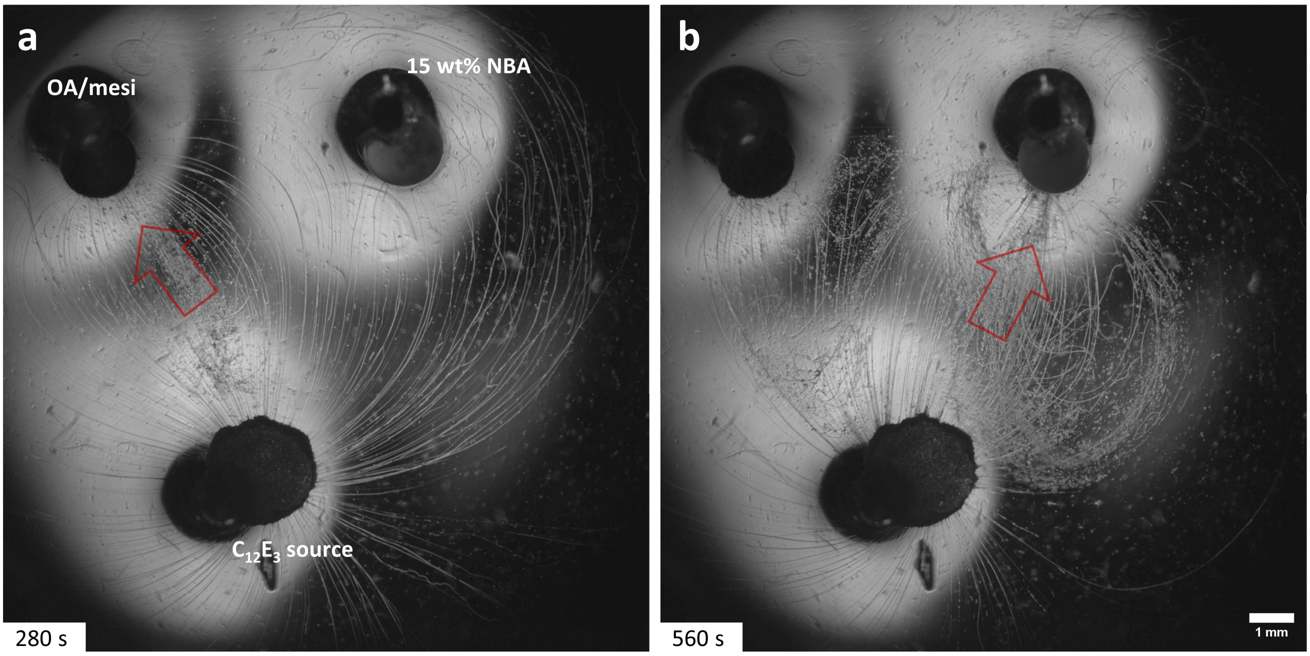

Figure S6. Optical microscopy images of a sample containing a $C_{12}E_3$ source droplet, an OA/mesi (1:1) and a 15 wt% NBA drain droplet. The OA/mesi drain is deposited at t = 0 s, the 15 wt% NBA drain at t = 75 s, and the system is exposed to UV at t = 92 s. The arrows indicate the main direction followed by the myelins. **(a)** At t = 280 s, the myelins still grow predominantly towards the OA/mesi drain despite the NBA drain being exposed to UV for nearly 200 s. **(b)** At t = 560 s, a large fraction of the myelins redirects towards the NBA drain. The scale bar represents 1 mm.

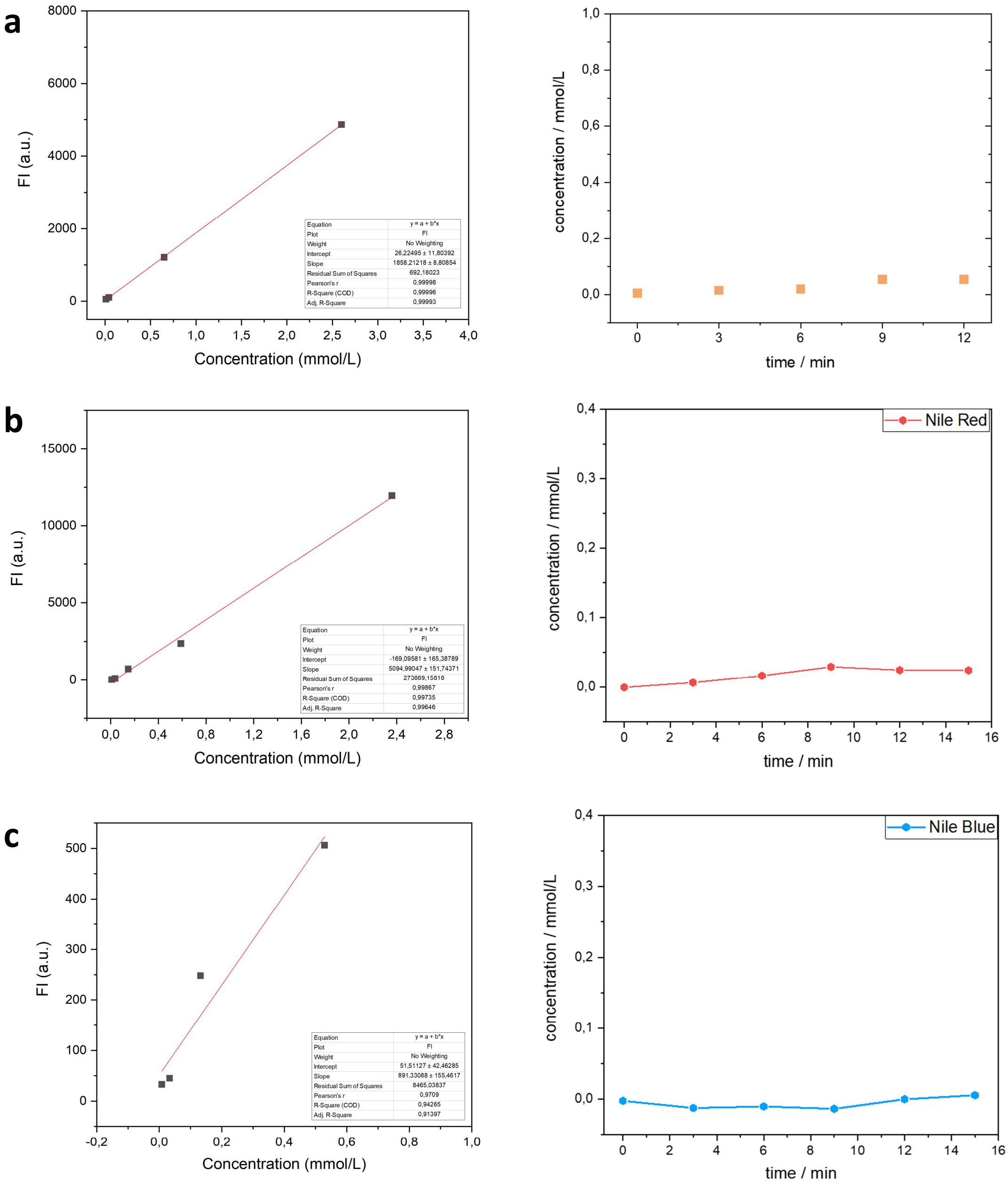

Figure S7. Test of dye leakage from the myelins. After deposition of a $C_{12}E_3$ droplet containing respectively 1 wt% neutral red (**a**), 1 wt% Nile Red (**b**) or 1 wt% Nile Blue (**c**) at the a/w interface, the dye content in the aqueous bulk is monitored by taking aliquots every 3 minutes and measuring their fluorescence spectra, from which the concentration is calculated based on the calibration curve (left).

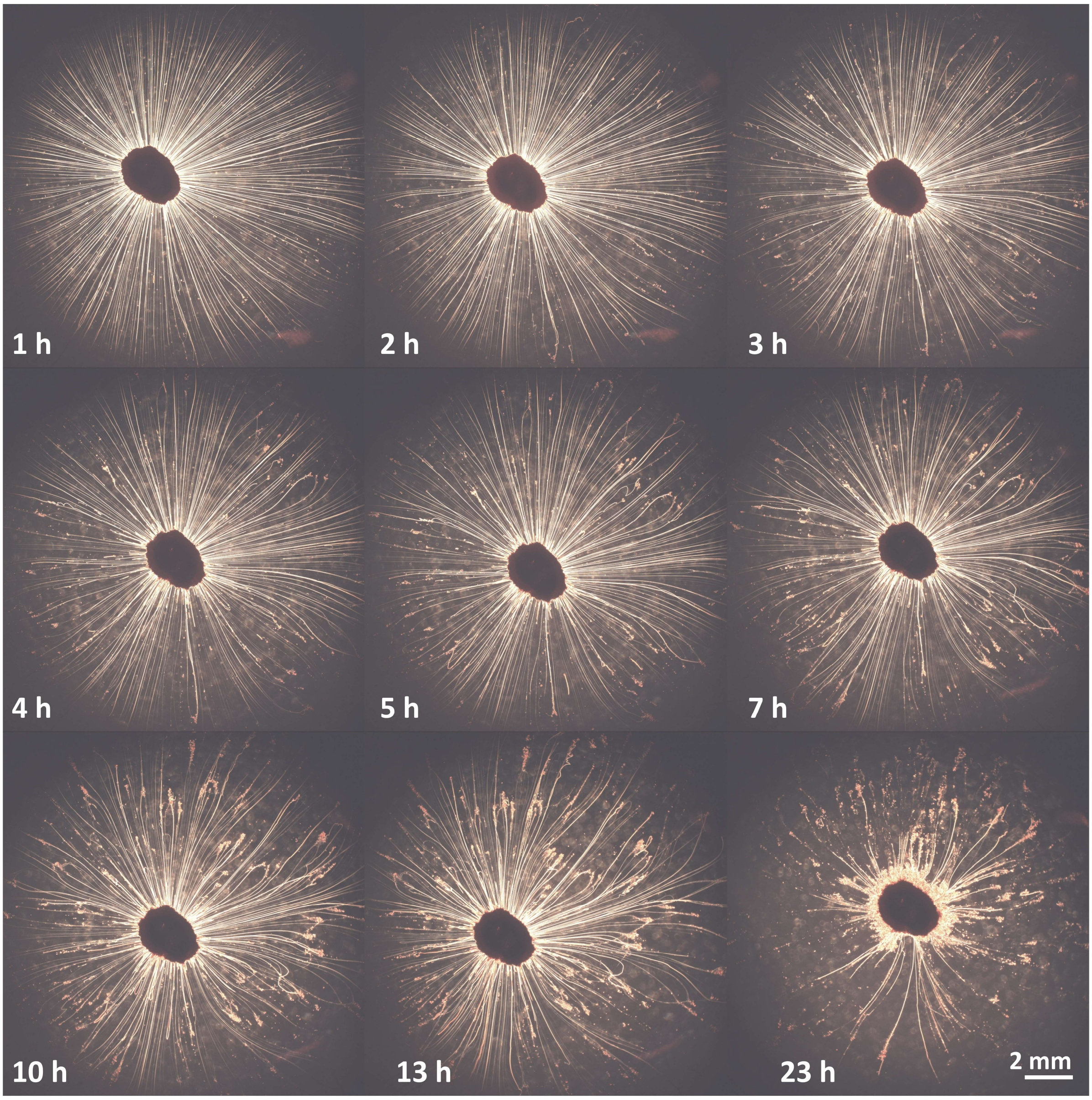

Figure S8. Optical microscopy recording of 1 µL $C_{12}E_3$ source droplet with 20 mg/mL Oil Red O, deposited on a 0.63 wt% sodium alginate solution.

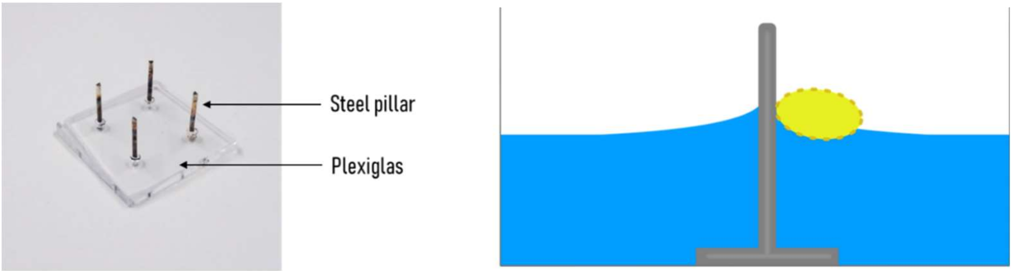

Figure S9. (left) Photograph of setup of steel pillars mounted in plexiglass base. (right) Scheme of pillar, with positive meniscus of aqueous medium. Via the "Cheerios effect" the source and drain droplets are kept in position at the pillars.[1]

Description of Movies 1 – 6

Movie 1 – corresponding to Fig. 1e: Optical microscopy recording of a 20 wt% NBA drain droplet (1.0 µL) deposited at an aqueous solution (0.63 wt% sodium alginate, 100 µM $C_{12}E_3$). The appearance of the purple ring in the movie indicates when the interface is exposed to UV.

Movie 2 – corresponding to Fig. 2c and Fig. S3: Optical microscopy recordings of $C_{12}E_3$ source droplet (2.0 µL) and drain droplet (2.0 µL, 10 – 20 wt% NBA) positioned at steel pillars. The appearance of the purple ring indicates the localized UV exposure of the drain droplets.

Movie 3 – corresponding to Fig. 3a and Fig. S5: Optical microscopy recordings of competition for myelins in a system with 4 drain droplets (2.0 µL) around a $C_{12}E_3$ source droplet (2.0 µL) in the center. All droplets are positioned at steel pillars. Left movie: 4 drains with similar NBA content (15 wt%); right movie: 4 drains with different NBA content (0, 5, 10 and 15 wt%). The appearance of the purple box indicates when the entire interface is exposed to UV.

Movie 4 – corresponding to Fig. 4a: Optical microscopy recording of a $C_{12}E_3$ source droplet (1.0 µL) placed at equidistance in between two 15 wt% NBA drains (2.0 µL); all droplets are positioned at steel pillars. The appearance of the purple ring indicates the localized UV exposure of the respective drain.

Movie 5 – corresponding to Fig. 4b and Fig. S6: Optical microscopy recordings of a $C_{12}E_3$ source droplet (left movie 2.0 µL; right movie 1.0 µL), a 1:1 OA/mesi drain (2.0 µL) and a 15 wt% NBA drain (2.0 µL), placed in different spatial conformations for two different experiments. All droplets are positioned at steel pillars. The appearance of the purple ring indicates the localized UV exposure of the NBA drain.

Movie 6 – corresponding to Fig. 5e: Optical microscopy recording of two $C_{12}E_3$ source droplets (1.0 µL) with Nile red (1 wt%, top left) and Nile blue (1 wt%, bottom right), and two 10 wt% NBA drain droplets (2.0 µL). The appearance of the UV icon in the scheme indicates when the top right drain droplet is locally exposed to UV.

References

(1) Vella, D.; Mahadevan, L. The "Cheerios Effect." *Am. J. Phys.* **2005**, *73* (9), 817–825.

(2) van der Weijden, A.; Winkens, M.; Schoenmakers, S. M. C.; Huck, W. T. S.; Korevaar, P. A. Autonomous Mesoscale Positioning Emerging from Myelin Filament Self-Organization and Marangoni Flows. *Nat. Commun.* **2020**, *11* (9), 4800.

(3) Winkens, M.; Korevaar, P. A. Self-Organization Emerging from Marangoni and Elastocapillary Effects Directed by Amphiphile Filament Connections. *Langmuir* **2022**, *38* (35), 10799–10809.

(4) Taylor, C. D.; Valkovska, D. S.; Bain, C. D. A Simple and Rapid Method for the Determination of the Surface Equations of State and Adsorption Isotherms for Efficient Surfactants. *Phys. Chem. Chem. Phys.* **2003**, *5* (21), 4885–4891.

(5) Bandi, M. M.; Akella, V. S.; Singh, D. K.; Singh, R. S.; Mandre, S. Hydrodynamic Signatures of Stationary Marangoni-Driven Surfactant Transport. *Phys. Rev. Lett.* **2017**, *119*, 264501.
